# Serum Sclerostin Correlates with ACPA Positivity in Post-Menopausal Women with Rheumatoid Arthritis: Data from a Cross-Sectional Case-Control Study in Greece

**DOI:** 10.31138/mjr.190425.eda

**Published:** 2026-03-27

**Authors:** Michail I. Krikelis, Dimitra Moschou, Konstantina Zoupidou, Evangelia Mole, Konstantinos Makris, Simeon Tournis, Sousana Gazi, Vassilios Nikolaou, Clio Mavragani, Efstathios Chronopoulos

**Affiliations:** 1 Laboratory for the Research of Musculoskeletal System “Th. Garofalidis”, Medical School, National and Kapodistrian University of Athens, KAT General Hospital, Athens, Greece;; 2 Rheumatologist, Larnaka, Cyprus;; 3 Attikon University Hospital, Rheumatology and Clinical Immunology Unit, 4th University Clinic of Internal Medicine, Athens, Greece;; 4 Rheumatology Department, KAT General hospital, Athens, Greece;; 5 Department of Biochemistry, KAT General Hospital, Athens, Greece;; 6 2nd University Clinic of Orthopaedic Surgery, Konstantopoulio General Hospital, Athens, Greece;; 7 Laboratory of Experimental Physiology, School of Medicine, National and Kapodistrian University of Athens, Athens, Greece;; 8 Laboratory for the Research of Musculoskeletal System “Th. Garofalidis”, Medical School, National and Kapodistrian University of Athens, KAT General Hospital, Athens, Greece

**Keywords:** rheumatoid arthritis, sclerostin, osteoporosis, sarcopenia, case-control study, Greece

## Abstract

**Introduction:**

Rheumatoid arthritis (RA) is a chronic inflammatory disease marked by bone loss, partly due to impaired Wnt signalling. The role of sclerostin, an endogenous Wnt inhibitor, in RA remains unclear.

**Methods:**

This 9-month cross-sectional case-control study included post-menopausal women recruited at a tertiary hospital in Athens, Greece. Participants underwent clinical assessment, DXA, radiographs, and blood tests. The aim was to assess serum sclerostin levels and their associations in RA.

**Results:**

A total of 97 RA patients and 45 controls were included. Mean age was 67 ± 7 years; median RA duration was 8 years; 40% were seropositive; 65% received bDMARDs. Median sclerostin levels did not differ significantly between groups (p=0.645). In RA patients, sclerostin levels were positively associated with ACPA (p=0.043). Notably, 27% of RA patients had extremely high sclerostin levels versus 11% of controls (p=0.035). Within this subgroup, high sclerostin was inversely associated with sarcopenia (p=0.020).

**Discussion:**

Although overall sclerostin levels were similar between groups, higher levels in seropositive RA suggest a possible link with ACPA.

## INTRODUCTION

Rheumatoid arthritis (RA) is a chronic inflammatory disease that primarily affects the small joints of the hands and feet, often leading to long-term functional impairment. As of 2020, an estimated 17.6 million people worldwide were living with RA, with a global prevalence of approximately 0.2%.^1^

The hallmark of the disease is synovial inflammation, which leads to the formation of pannus—a thickened synovial tissue that perpetuates inflammation and contributes to joint and bone damage.^2^

Bone loss in RA occurs at three levels: local bone erosions, periarticular demineralisation, and generalised osteopenia or osteoporosis.^3^ This bone depletion is driven by chronic systemic and local joint inflammation, as well as by long-term corticosteroid use.^4^ Clinically, this is highly relevant, as up to 60% of RA patients develop osteoporosis, and 13% sustain osteoporotic vertebral fractures.^5^ Furthermore, muscle wasting (sarcopenia), driven by inflammation and reduced mobility, worsens bone fragility in RA.^6^

The RANK/RANKL/OPG pathway is central to bone resorption in RA, promoting osteoclast formation in response to inflammatory cells and cytokines like TNF-α and IL-6.^7^ However, bone formation is also impaired. The Wnt signalling pathway, which regulates osteoblast activity, is disrupted in RA. This disruption is largely due to inhibitors like Dkk-1, produced by inflammatory cells, which block Wnt signalling and osteoblast maturation.^8^

Sclerostin, another Wnt pathway inhibitor, is mainly produced by osteocytes in healthy bone and helps regulate bone remodelling in response to mechanical loading.^9^ Yet, in arthritis models, synovial fibroblasts have been shown to produce sclerostin when exposed to TNF-α, suggesting it may also contribute to bone loss in RA.^10^ Still, clinical studies have yielded conflicting results regarding circulating sclerostin levels and their significance in RA.^11^ Similarly, studies on anti-sclerostin therapies in animal models have not consistently shown benefit in preventing or reversing bone erosions.^12,13^

To explore this further, we conducted a case-control study to examine serum sclerostin levels in RA patients versus healthy controls and to assess disease-specific factors that may influence sclerostin levels. To reduce confounding, we selected a homogenous study population of post-menopausal women without significant comorbidities. This rigorous selection strengthens the validity of our findings and represents a methodological novelty among similar studies.

## METHODS

### Study Population

This cross-sectional case-control study was conducted between January and September 2022 at the outpatient rheumatology clinic of KAT General Hospital, Athens, Greece. A convenience sample of post-menopausal women with RA was recruited. Age-matched healthy controls were randomly selected from individuals presenting with non-inflammatory musculoskeletal conditions (e.g., osteoarthritis, mechanical back pain). All participants provided written informed consent. The study protocol was approved by the hospital’s scientific and ethics committee (decision no. 5697 / 04-05-2022).

### Eligibility Criteria

RA was diagnosed per the 2010 ACR/EULAR criteria.^14^ Only post-menopausal women under ongoing RA treatment were included. Exclusion criteria included: untreated or newly diagnosed RA, male sex, pre-menopausal status, major comorbidities (e.g., renal failure, malignancy), mobility impairments, joint deformities, or prior hip arthroplasty.

Controls were free of chronic inflammatory or severe systemic diseases.

### Clinical and Laboratory Assessment

Participants underwent structured clinical assessments capturing:

Demographics: age, height, weight, BMI, years post-menopause.
RA characteristics: disease duration, seropositivity (RF, ACPA), inflammatory markers (ESR, CRP), DAS28-ESR, HAQ-DI, and treatment regimens.
Bone health: history of fragility fractures, osteoporosis treatment.

### Bone and Muscle Evaluation

All subjects underwent whole-body DXA (Lunar Prodigy Pro, GE) by a single blinded technician. BMD was measured at the lumbar spine, femoral neck, and total hip. Sarcopenia was diagnosed per EWGSOP2 criteria^16^ using:

- Appendicular Skeletal Muscle Index (ASMI),
- Grip strength (JAMAR digital hand dynamometer),
- Physical performance (Short Physical Performance Battery),
- Daily physical activity (IPAQ).

All evaluations were performed by a blinded, trained occupational therapist.

Osteoporosis was defined as a T-score ≤ −2.5 and/or ongoing anti-osteoporotic therapy (excluding calcium/vitamin D). RA patients also had hand/foot X-rays to calculate the modified Sharp Score (mSS).^17^

### Biochemical Analysis

Fasting blood samples were collected, centrifuged, and stored at −80°C. Serum sclerostin was measured using a Human SOST ELISA Kit (PicoKine, BOSTER) on a Cobas e411 analyser (Roche). The assay had <4.0% analytical imprecision.

“High” sclerostin was defined as >100 pg/ml; “extremely high” as >1000 pg/ml, based on manufacturer thresholds for reliable optical density (OD) readings.

### Statistical Analysis

Comparisons were made between RA patients and controls, as well as within the RA group. Normality was assessed with the Kolmogorov–Smirnov test. Normally distributed data are reported as mean ± SD; skewed data as median (min–max).

Group differences were tested using ANOVA or Kruskal–Wallis for continuous variables and chi-square for categorical data. Associations are presented as odds ratios (OR) with 95% confidence intervals (CI). A p-value <0.05 was considered statistically significant. Analyses were conducted using IBM SPSS v24.

## RESULTS

A total of 97 RA patients and 45 controls were enrolled.

### Study Population (Table 1)

The mean age of RA patients was 67 ± 7 years, and the mean BMI was significantly higher than in controls (29.3 vs 26.5 kg/m^2^, *p*=0.003), reflecting typical RA-associated metabolic changes and reduced physical activity.

**Table 1.** Study variables and comparisons between RA patients and controls.

	**Patients (n=97)**	**Controls (n=45)**	**p-value**

**Demographics**			

Age (years) Mean (±SD)	67 (±7)	66 (±10)	0.428

BMI (kg/m^2^) Mean (±SD)	29.3 (±5.3)	26.5 (±4.9)	**0.003**

Years post-menopause Mean (±SD)	21 (±10)	22 (±7)	0.428

**Disease parameters**			

Disease years			-
Median (min, max)	8 (1–10)	NA	

Anti-CCP n (%)	37 (41%)	NA	-

RF n (%)	40 (43%)	NA	-

**Disease Treatment**			
Glucocorticoids (≤ 7 mg of prednisolone equivalent perday) n (%)	50 (52% )		
MTX n (%)	47 (49%)	NA	-
LEF n (%)	24 (25%)		
HCQ n (%)	7 (7%)		
bDMARD n (%)	62 (64%)		

ESR (mmHg) Median (min, max)	21 (2–94)	15 (5–48)	**<0.001**

CRP (mg/dl) Median (min, max)	0.33 (0.04–10.00)	0.30 (0.30–1.92)	**<0.001**

DAS 28-ESR			-
Mean (±SD)	3.77 (±1.66)	NA	

Disease activity			
High disease activity n (%)	26 (27%)		
Moderate disease activity n (%)	16 (17%)	NA	-
Low disease activity n (%)	31 (32%)		
Remission n (%)	23 (24%)		

HAQ-DI			-
Median (min, max)	0.75 (0–2.25)	NA	

**Functional impairment**			
Functional remission n (%)	57 (60%)		
Moderate disability n (%)	33 (35%)	NA	-
Severe disability n (%)	5 (5%)		

Modified Sharp Score (mSS)			-
Median (min, max)	18 (5–156)	NA	

**Osteoporosis parameters**			

Osteoporosis n (%)	74 (80%)	17 (39%)	**<0.001**

**DXA measurements median (min, max)**			
BMD LS (g/cm^2^)	1.059 (0.796–1.684)	0.985 (0.735–1.567)	0.655
BMD FN (g/cm^2^)	0.792 (0.573–1.051)	0.809 (0.598–1.114)	0.714
BMD TH (g/cm^2^)	0.848 (0.620–1.226)	0.866 (0.563–1.207)	0.591
TBS	1.290 (0.835–1.615)	1.297 (1.089–1.569)	0.274
SI	1.4 (0.8–2.1)	1.4 (0.7–2.3)	0.510
CSMI (mm^4^)	7.7 (4.0–16.6)	8.1 (4.7–11.5)	0.643
CSA (mm^2^)	118 (86–169)	121 (87–168)	0.983

**Treatment for osteoporosis**			
Calcium supplement n (%)	57 (59%)	7 (16%)	<0.001
Vitamin D supplement n (%)	63 (65%)	12 (27%)	**<0.001**
Bisphosphonates n (%)	40 (41%)	6 (13%)	**<0.001**
Denosumab n (%)	7 (7%)	1 (2%)	0.230
Teriparatide n (%)	0	0	-
Romosozumab n (%)	0	0	-
Fracture n (%)	37 (38%)	9 (20%)	**0.032**
Hip fracture n (%)	4 (4%)	2 (4%)	1.000
Vertebral fracture n (%)	8 (8%)	3 (7%)	0.743
Other fracture n (%)	30 (31%)	5 (11%)	**0.011**

**Sarcopenia parameters**			

Sarcopenia n (%)	33 (38%)	0.30 (0.30–1.92)	<0.001

Severe sarcopenia n (%)	12 (14%)	0	**0.009**

ASMI (kg/m^2^_)_			
Median (min, max)	6.1 (4.2–8.0)	6.6 (4.4–12.3)	**0.026**

Hand grip strength (kg)			
Median (min, max)	15 (0–85)	27 (2–60)	**<0.001**

Physical performance (SPPB)			
Median (min, max)	8 (2–12)	12 (8–16)	**<0.001**

IPAQ			
Median (min, max)	1323 (0–8370)	1500 (120–6780)	0.240

Physical activity			
Low (IPAQ<600) n (%)	29 (31%)	6 (13%)	
Moderate (IPAQ 600–3000) n (%)	41 (43%)	29 (64%)	0.033
High (IPAQ>3000) n (%)	24 (26%)	10 (23%)	

**Osteosarcopenia**			

Osteosarcopenia n (%)	29 (32%)	0	**<0.001**

Bone metabolism			

Ca (mg/dL) Median (min, max)	9.3 (8.4–10.7)	9.3 (8.6–10.3)	0.790

Alb (gr/dL) Median (min, max)	4.2 (3.4–5.3)	4.4 (0.4–4.9)	**<0.001**

P (mg/dL)			
Median (min, max)	3.5 (1.9–4.6)	3.7 (2.9–4.4)	**0.040**

Mg (mg/dL) Median (min, max)	1.9 (1.5–4.7)	2.2 (1.8–4.2)	**<0.001**

Cr (mg/dL) Median (min, max)	0.74 (0.50–1.87)	0.71 (0.60–1.02)	0.260

ALP (IU/L)			
Median (min, max)	67 (30–166)	67 (33–100)	0.629

BALP (ng/mL) Median (min, max)	10.8 (6.8–23.1)	16.5 (9.8–43.0)	**0.022**

PTH (pg/mL) Median (min, max)	49.9 (2.0–148.0)	42.4 (12.3–80.0)	0.005

25(OH)D3 (ng/mL)			
Median (min, max)	29.7 (10.7–65.0)	23.7 (3.7–53.6)	**0.011**

TSH (µU/mL) Median (min, max)	1.2 (0.1–19.6)	1.4 (0.2–17.1)	0.623

OCN (mg/L) Median (min, max)	14.3 (3.6–40.4)	18.6 (7.5–36.4)	**0.004**

CTX (pg/mL) Median (min, max)	0.25 (0.03–0.84)	0.30 (0.10–0.66)	**0.025**

IGF-I (ng/ml) Median (min, max)	92.6 (28.9–262.3)	119.9 (51.6–200.0)	**0.009**

Serum sclerostin			

Serum sclerostin (pmol/lt)			
Median (min, max)	70.6 (3.1–5841.1)	67.2 (2.3–1821.7)	0.645

For continuous variables with a normal distribution data are presented as means (± SD), for continuous variables with a non-normal distribution data are presented as median (minimum, maximum), whereas for non-continuous variables data are presented as n (%).

BMI; Body mass index; Anti-CCP; anti-cyclic citrullinated peptide antibody; RF; Rheumatoid factor; MTX; Methotrexate; LEF; Leflunomide; HCQ; Hydroxychloroquine; bDMARD; biological disease-modifying antirheumatic drugs; ESR; Erythrocyte Sedimentation Rate; CRP; C-reactive protein; DAS28-ESR; disease activity score in 28 joints-erythrocyte sedimentation rate; HAQ-DI; Health Assessment Questionnaire Disability Index; DXA; dual-energy X-ray absorptiometry; BMD; bone mineral density; LS; lumbar spine; FN; femoral neck; TH; total hip; TBS; trabecular bone score; SI; strength index; CSMI; cross-sectional moment of inertia; CSA; cross-sectional area; ASMI; skeletal muscle mass index; SPPB; Short Physical Performance Battery; IPAQ; International Physical Activity Questionnaire; Ca; calcium; Alb; albumin; P; Phosphorus; Mg; magnesium; Cr; creatinine; ALP; alkaline phosphatase; BALP; bone alkaline phosphatase; PTH; parathyroid hormone; 25(OH)D3; 25-hydroxyvitamin D3; TSH; thyroid stimulating hormone; OCN; osteocalcin; CTX; cross-linked C-telopeptide of type I collagen; IGF-1; insulin-like growth factor 1; NA: not applicable. Statistical significance if p<0.05.

Median disease duration was 8 (1–10) years, and 40% were seropositive. All patients were on treatment: 52% on low-dose corticosteroids (≤7 mg prednisolone/day), 35% on cDMARDs alone, and 65% on cDMARDs + bDMARDs.

The mean DAS28-ESR score was 3.8 ± 1.7; 27% were in remission and 32% had low disease activity. Median HAQ-DI was 0.75 (0–2.25), with 34% achieving functional remission. Radiographic damage was present in 59% (median mSS: 18 [5–156]).

Osteoporosis was significantly more prevalent in RA patients than in controls (80% vs 39%, *p*<0.001), with an odds ratio (OR) of 6.1 (95% CI: 2.8–13.6). Fragility fractures were reported in 38% of patients, and 48% were on anti-osteoporotic treatment. Despite this, BMD did not significantly differ between groups, reflecting effective osteoporosis management.

Sarcopenia was detected in 38% of RA patients, but in none of the controls (*p*<0.001; OR = 27, 95% CI: 3.6– 208.4). Severe sarcopenia was also significantly more common in RA patients (14% vs 0%, *p*=0.009; OR = 7.1, 95% CI: 0.9–56.7).

Median serum sclerostin levels did not differ significantly between groups (70.6 vs 67.2 pmol/L, *p*=0.645). However, this association is reversed if the outlier values (serum sclerostin >1,000 pmol/L) are discarded (p=0.026).

### Sclerostin Associations in RA (Table 2)

In RA patients, serum sclerostin levels showed no significant association with age, disease duration, disease activity, radiographic damage, treatment type, osteoporosis, sarcopenia, or IGF-I levels. The only significant association was with ACPA positivity (*p*=0.043).

**Table 2.** Correlation of serum sclerostin with several parameters among RA patients and controls.

	**Patients (n=97)**	**Controls (n=45)**

**Demographics**		

Age (years)	p=0.527	p=0.607

BMI (kg/m^2^)	p=0.701	p=0.984

Years post-menopause	p=0.398	p=0.424

Disease parameters		

Disease years	p=0.500	NA

Anti-CCP	p=0.043	NA

RF	p=0.433	NA

**Disease Treatment**		
Glucocorticoids (≤ 7 mg of prednisolone equivalent per day)	p=0.249	NA
MTX	p=0.364	
LEF	p=0.071	
HCQ	p=0.351	
bDMARD	p=0.442	

ESR (mmHg)	p=0.855	p=0.355

CRP (mg/dl)	p=0.285	p=0.037

DAS 28-ESR	p=0.584	NA

**Disease activity**		
High disease activity		
Moderate disease activity	p=0.390	NA
Low disease activity		
Remission		

HAQ-DI	p=0.490	NA

Functional impairment		
Functional remission		
Moderate disability	p=0.999	NA
Severe disability		

Modified Sharp Score (mSS)	p=0.550	NA

**Osteoporosis parameters**		

Osteoporosis	p=0.936	p=0.189

**DXA measurements**		
BMD LS (g/cm^2^)	p=0.359	p=0.577
BMD FN (g/cm^2^)	p=0.179	p=0.629
BMD TH (g/cm^2^)	p=0.364	p=0.976
TBS	p=0.411	p=0.128
SI	p=0.026	p=0.149
CSMI (mm^4^)	p=0.035	p=0.558
CSA (mm^2^)	p=0.046	p=0.784

**Treatment for osteoporosis**		
Calcium supplement	p=0.062	p=0.123
Vitamin D supplement	p=0.487	p=0.381
Bisphosphonates	p=0.652	p=0.107
Denosumab	p=0.573	p=1.000
Teriparatide	-	-
Romosozumab	-	-

**Fracture**	p=0.247	p=0.511
Hip fracture	p=0.934	p=0.848
Vertebral fracture	p=0.522	p=0.259
Other fracture	p=0.096	p=0.471

**Sarcopenia parameters**		

Sarcopenia	p=0.985	-

Severe sarcopenia	p=0.079	-

ASMI (kg/m^2^)	p=0.989	p=0.489

Hand grip strength (kg)	p=0.425	p=0.168

Physical performance (SPPB)	p=0.789	p=0.841

IPAQ	p=0.949	p=0.592

Physical activity Low (IPAQ<600)		
Moderate (IPAQ 600–3000) High (IPAQ>3000)	p=0.811	p=0.398

**Osteosarcopenia**		

Osteosarcopenia	p=0.861	-

**Bone metabolism**		

Ca (mg/dL)	p=0.211	p=0.804

Alb (gr/dL)	p=0.186	**p=0.018**

P (mg/dL)	p=0.500	p=0.921

Mg (mg/dL)	p=0.590	p=0.483

Cr (mg/dL)	p=0.831	p=0.688

ALP (IU/L)	p=0.960	p=0.657

BALP (ng/mL)	p=0.169	p=0.343

PTH (pg/mL)	p=0.458	p=0.588

25(OH)D3 (ng/mL)	p=0.114	p=0.968

TSH (µU/mL)	p=0.179	p=0.707

OCN (mg/L)	p=0.165	p=0.132

CTX (pg/mL)	p=0.977	p=0.958

IGF-I (ng/ml)	p=0.630	p=0.773

BMI; Body mass index; Anti-CCP; anti-cyclic citrullinated peptide antibody; RF; Rheumatoid factor; MTX; Methotrexate; LEF; Leflunomide; HCQ; Hydroxychloroquine; bDMARD; biological disease-modifying antirheumatic drugs; ESR; Erythrocyte Sedimentation Rate; CRP; C-reactive protein; DAS28-ESR; disease activity score in 28 joints-erythrocyte sedimentation rate; HAQ-DI; Health Assessment Questionnaire Disability Index; DXA; dual-energy X-ray absorptiometry; BMD; bone mineral density; LS; lumbar spine; FN; femoral neck; TH; total hip; TBS; trabecular bone score; SI; strength index; CSMI; cross-sectional moment of inertia; CSA; cross-sectional area; ASMI; skeletal muscle mass index; SPPB; Short Physical Performance Battery; IPAQ; International Physical Activity Questionnaire; Ca; calcium; Alb; albumin; P; Phosphorus; Mg; magnesium; Cr; creatinine; ALP; alkaline phosphatase; BALP; bone alkaline phosphatase; PTH; parathyroid hormone; 25(OH)D3; 25-hydroxyvitamin D3; TSH; thyroid stimulating hormone; OCN; osteocalcin; CTX; cross-linked C-telopeptide of type I collagen; IGF-1; insulin-like growth factor 1; NA: not applicable.

Statistical significance if p<0.05

**Table 3.** Multiple logistic regression analysis for the correlation of serum sclerostin within the RA patient arm.

**High sclerostin values (>100 mmol/lt)** **Model fit p=0.039, R^2^ 49%**	**Patients (n=29)**
Age (years)	**p=0.045**
BMI (kg/m^2^)	p=0.895
Years post-menopause	p=0.090
Disease years	p=0.393
Anti-CCP	**p<0.001**
RF	**p=0.003**
Disease treatment with low-dose corticosteroids	p=0.219
Disease Treatment with bDMARD	p=0.398
ESR (mmHg)	p=0.075
CRP (mg/dl)	p=0.129
DAS 28-ESR	p=0.897
HAQ-DI	p=0.709
Modified Sharp Score (mSS)	p=0.224
Osteoporosis	**p=0.036**
Sarcopenia	p=0.778
ASMI (kg/m^2^)	p=0.645
TBS	p=0.177
eGFR	p=0.573
**Extremely high sclerostin values (>1,000 mmol/lt)** **Model fit p<0.001, R^2^ 67%**	**Patients (n=25)**
Age (years)	p=0.999
BMI (kg/m^2^)	p=0.999
Years post-menopause	p=0.999
Disease years	p=0.998
Anti-CCP	**p<0.001**
RF	**p<0.001**
Disease treatment with low-dose corticosteroids	p=0.999
Disease Treatment with bDMARD	p=0.999
ESR (mmHg)	**p<0.001**
CRP (mg/dl)	**p<0.001**
DAS 28-ESR	**p=0.045**
HAQ-DI	p=0.998
Modified Sharp Score (mSS)	**p<0.001**
Osteoporosis	p=1.000
Sarcopenia	p=0.999
ASMI (kg/m^2^)	p=1.000
TBS	p=1.000
eGFR	p=0.998

BMI, Body mass index; Anti-CCP, anti-cyclic citrullinated peptide antibody; RF, Rheumatoid factor; MTX, Methotrexate; LEF, Leflunomide; HCQ, Hydroxychloroquine; bDMARD, biological disease-modifying antirheumatic drugs; ESR, Erythrocyte Sedimentation Rate; CRP, C-reactive protein; DAS28-ESR, disease activity score in 28 joints-erythrocyte sedimentation rate; HAQ-DI, Health Assessment Questionnaire Disability Index; DXA, dual-energy X-ray absorptiometry; BMD, bone mineral density; LS, lumbar spine; FN, femoral neck; TH, total hip; TBS, trabecular bone score; SI, strength index; CSMI, cross-sectional moment of inertia; CSA, cross-sectional area; ASMI, skeletal muscle mass index; SPPB, Short Physical Performance Battery; IPAQ, International Physical Activity Questionnaire; Ca, calcium; Alb, albumin; P, Phosphorus; Mg, magnesium; Cr, creatinine; ALP, alkaline phosphatase; BALP, bone alkaline phosphatase; PTH, parathyroid hormone; 25(OH)D3, 25-hydroxyvitamin D3; TSH, thyroid stimulating hormone; OCN, osteocalcin; CTX, cross-linked C-telopeptide of type I collagen; IGF-1, insulin-like growth factor 1; NA: not applicable.

Statistical significance if p<0.05

In multivariate logistic regression, ACPA positivity was the sole predictor of high sclerostin levels (>100 pmol/L), with an OR of 2.8 (95% CI: 1.1–7.2, *p*=0.030). The model had good fit (*p*=0.039, R^2^=49%).

Notably, 27% of RA patients (n=25) had extremely high sclerostin levels (>1,000 pmol/L) versus 11% of controls (*p*=0.035). Within this subgroup, no differences were found in disease characteristics, osteoporosis, or general sarcopenia rates. In multivariate logistic regression, ACPA positivity was an important predictor of extremely high sclerostin levels (>1000 pmol/L), with an OR of 2.5 (95% CI: 0.9–6.6, *p*=0.06). Other predictors of extremely high sclerostin levels were the persistent inflammatory disease, as expressed by increased ESR (p<0.001), increased CRP (p<0.001) and increased DAS28-ESR (p=0.045). Increased modified sharp score was also associated with extremely high serum sclerostin levels (p<0.001). The model had good fit (*p*<0.001, R^2^=67%).

## DISCUSSION

In this cross-sectional case-control study, we assessed serum sclerostin levels in post-menopausal women with rheumatoid arthritis (RA) compared to healthy controls and examined their associations with disease characteristics, bone metabolism markers, bone mineral density (BMD), and parameters of sarcopenia.

Our RA cohort consisted of a carefully selected group of post-menopausal women with longstanding disease, all of whom were under treatment and receiving osteoporosis management. While this stringent selection minimises confounding factors affecting sclerostin levels, it may limit the broader representativeness of the findings. Nevertheless, this population reflects the typical outpatient demographic of a tertiary hospital in Greece, allowing for cautious generalisation.

The primary finding of the study is that serum sclerostin levels did not differ significantly between RA patients and healthy controls. This result aligns with some existing studies, though others have reported elevated sclerostin levels in RA patients. A recent meta-analysis suggested that RA patients tend to exhibit increased sclerostin concentrations; however, the considerable heterogeneity across studies precludes definitive conclusions.^19^ For instance, Boman et al. reported increased sclerostin in early RA (20), while an Egyptian cohort with longstanding RA also demonstrated elevated levels.^21^ Conversely, other studies—including an additional Egyptian cohort and the French study by Paccou et al.—found no significant differences.^22,23^ In our cohort, the lack of difference appears to be influenced by the presence of extremely high sclerostin values in both groups, more commonly in RA patients. When these outliers were excluded, the contrast between patients and controls became more apparent. We hypothesise that these aberrant values—particularly among RA patients—may reflect a disease-specific pathophysiologic process, though the mechanism remains unclear.

Importantly, serum sclerostin in RA patients showed no association with demographic characteristics, disease activity, structural damage, functional status, treatments, bone metabolism markers, osteoporosis, or sarcopenia. The sole significant correlation was with ACPA positivity: patients positive for anti-citrullinated protein antibodies (ACPA) had significantly higher sclerostin levels than ACPA-negative individuals. This finding suggests a possible role for ACPA in modulating sclerostin expression, potentially contributing to RA-related bone loss.

ACPA are known to be implicated in systemic bone loss in early RA, as they promote osteoclastogenesis by binding to myeloid precursor cells.^24,25^ They also contribute to sustained inflammation by inducing TNF-α secretion from activated macrophages, which in turn stimulates sclerostin production by synovial fibroblasts.^26^ Whether ACPA directly enhance sclerostin secretion by juxta-articular or systemic osteocytes remains uncertain. While interactions between ACPA and synovial osteocytes have been documented,^27,28^ their effects on osteocytes at extra-articular skeletal sites require further investigation.

Despite its cross-sectional design, our study benefits from robust methodology, including a well-defined patient cohort and control group, strict inclusion/exclusion criteria, and optimised laboratory procedures.

These strengths enhance the reliability of our findings. The central result—that serum sclerostin levels do not significantly differ between post-menopausal women with RA and healthy controls—adds valuable nuance to the current literature.

The secondary observation of extremely elevated sclerostin levels in a subset of RA patients introduces a novel line of inquiry into the interplay between ACPA and osteocytes. This phenomenon raises the hypothesis that ACPA-driven overproduction of sclerostin may contribute to the systemic bone loss seen in RA. Experimental studies are needed to elucidate this mechanism and to assess its clinical relevance.

Building on our findings and those in the literature, we propose that sclerostin may follow a bimodal distribution in RA. In early disease, elevated sclerostin is likely driven by high disease activity and the surge of pro-inflammatory cytokines.^20^ These cytokines may induce sclerostin production by synovial fibroblasts, chondrocytes, and subchondral osteocytes within the inflamed joint environment. Systemically, this contributes to generalised bone loss, while locally, sclerostin’s role appears dual: inhibiting osteoblast differentiation and promoting erosions, yet potentially protecting cartilage from degradation through poorly understood mechanisms.^10–13^

In established RA, under immunomodulatory therapy, persistently elevated serum sclerostin levels may reflect ongoing synovium–bone crosstalk. Chronic inflammation within the pannus may continue to drive sclerostin expression, thereby sustaining both local bone erosions and systemic bone loss. This pathological interaction may also be mediated, at least in part, by the presence of autoantibodies.

These observations suggest that anti-sclerostin therapy, such as romosozumab, could offer dual therapeutic benefits in RA: mitigating systemic osteoporosis and potentially promoting the repair of marginal bone erosions when used as an adjunct to conventional immunosuppressive treatment. Further clinical and experimental studies are needed to explore the therapeutic potential of sclerostin inhibition in the context of RA.
